# Regulation of Long Non-coding RNA KCNQ1OT1 Network in Colorectal Cancer Immunity

**DOI:** 10.3389/fgene.2021.684002

**Published:** 2021-09-22

**Authors:** Junjie Liu, Wei Lv, Shuling Li, Jingwen Deng

**Affiliations:** ^1^ Guangzhou Panyu Central Hospital, Guangzhou, China; ^2^ Guangdong Provincial Hospital of Chinese Medicine, Guangzhou, China

**Keywords:** long non-coding RNA, KCNQ1OT1, ceRNA network, colorectal cancer, immunity

## Abstract

Over the past few decades, researchers have become aware of the importance of non-coding RNA, which makes up the vast majority of the transcriptome. Long non-coding RNAs (lncRNAs) in turn constitute the largest fraction of non-coding transcripts. Increasing evidence has been found for the crucial roles of lncRNAs in both tissue homeostasis and development, and for their functional contributions to and regulation of the development and progression of various human diseases such as cancers. However, so far, only few findings with regards to functional lncRNAs in cancers have been translated into clinical applications. Based on multiple factors such as binding affinity of miRNAs to their lncRNA sponges, we analyzed the competitive endogenous RNA (ceRNA) network for the colorectal cancer RNA-seq datasets from The Cancer Genome Atlas (TCGA). After performing the ceRNA network construction and survival analysis, the lncRNA KCNQ1OT1 was found to be significantly upregulated in colorectal cancer tissues and associated with the survival of patients. A KCNQ1OT1-related lncRNA-miRNA-mRNA ceRNA network was constructed. A gene set variation analysis (GSVA) indicated that the expression of the KCNQ1OT1 ceRNA network in colorectal cancer tissues and normal tissues were significantly different, not only in the TCGA-COAD dataset but also in three other GEO datasets used as validation. By predicting comprehensive immune cell subsets from gene expression data, in samples grouped by differential expression levels of the KCNQ1OT1 ceRNA network in a cohort of patients, we found that CD4^+^, CD8^+^, and cytotoxic T cells and 14 other immune cell subsets were at different levels in the high- and low-KCNQ1OT1 ceRNA network score groups. These results indicated that the KCNQ1OT1 ceRNA network could be involved in the regulation of the tumor microenvironment, which would provide the rationale to further exploit KCNQ1OT1 as a possible functional contributor to and therapeutic target for colorectal cancer.

## Introduction

Colorectal cancer (CRC) is a common malignant cancer and is the second-highest contributor to the worldwide incidence of cancer-related deaths (Miller et al., 2019). CRC develops sporadically from some inflammatory bowel diseases or hereditary cancer syndromes. The development of colorectal cancer is based on the adenoma-carcinoma sequence. So far, the molecular mechanism of the adenoma-carcinoma sequence has been only partly identified. CRC prognosis depends on factors related to the patient and treatment. The expertise of the treatment team is one of the most important determinants of the outcome. Early detection of CRC cells and cancer precursor cells significantly reduce morbidity and improve patient prognosis (Sung et al., 2021).

The mortality of colorectal cancer can be effectively reduced by screening for the cancer. The most common screening procedures include flexible sigmoidoscopy, double-contrast barium enema, fecal occult blood tests, and colonoscopy (Bibbins-Domingo et al., 2016). There is no consensus regarding which screening method is the best, and it appears that no one test is better than the other. Risk, cost, and effectiveness are the main factors to be considered when discussing different options (Issa and Noureddine, 2017). Undoubtedly, a complete colonoscopy has the advantages of allowing the entire colon to be assessed, material for a biopsy to be collected, and a polypectomy to be carried out all within the same examination time; however, it also has disadvantages of higher costs as well as risks, discomfort and inconvenience for the patient being examined. Therefore, it is for all practical purposes necessary to develop effective biomarkers of CRC for applications in screening, diagnosis and prognosis.

Most of the RNA transcribed from the human genome does not encode for proteins. Some of these non-coding RNAs (ncRNAs) have been found to dysregulate the normal expression of genes, including tumor suppressor genes and oncogenes. Therefore, ncRNAs are considered to be new promising targets for studying tumorigenesis. ncRNAs include long non-coding RNAs (lncRNAs), microRNAs (miRNAs), circular RNAs, and small interfering RNAs (siRNAs). An miRNA is a highly conserved non-coding RNA approximately 21–24 nucleotides in length, and interacts with target mRNAs to regulate gene expression. Some miRNAs have been reported to be involved in the occurrence and development of cancer (Hayes et al., 2014), and miRNA-based therapeutics have in fact reached the stage of clinical development (Toden et al., 2021). In recent years, the importance of lncRNA has gradually become recognized. An lncRNA is essentially an ncRNA with more than 200 base pairs. So far, some lncRNAs have been shown to play a major regulatory role in genetic regulation. Recent studies have shown multiple roles for lncRNAs in tumorigenesis (de Oliveira et al., 2019). The competitive endogenous RNA (ceRNA) hypothesis is related to lncRNAs and miRNAs, proposed by Salmena and others (Salmena et al., 2011), has been described as the “Rosetta Stele”, used to decode the RNA language in order to regulate RNA crosstalk and regulate biological functions. Many studies have shown that miRNA-mediated ceRNA regulation plays a crucial role in the occurrence and development of cancer (de Oliveira et al., 2019). Long non-coding RNA KCNQ1 opposite strand/antisense transcript one gene (KCNQ1OT1) were markedly upregulated in gastric cancer tissues and cells (Zhong et al., 2021). High expression of lncRNA KCNQ1OT1 was significantly related to poor survival in patients with CRC in a pan-cancer meta-analysis. The upregulation of KCNQ1OT1 in CRC tissues and cell lines was also confirm the important role of KCNQ1OT1 in CRC (Lin et al., 2021). In our current work, we investigated the KCNQ1OT1 by mining the CRC RNA-Seq dataset in The Cancer Genome Atlas (TCGA), tested its prognostic potential in a CRC cohort and constructed the KCNQ1OT1-related lncRNA-miRNA-mRNA ceRNA network. We aimed to provide the rationales to the further exploitation of KCNQ1OT1 as a possible functional contributor to and therapeutic target for CRC.

## Materials and Methods

### Differentially Expressed Genes From TCGA RNA-Seq Data

RNA-seq data of a colorectal cancer cohort (TCGA-COAD) was downloaded with gdc-client (version 1.3.0) from the data portal of TCGA (the dbGaP accession: phs000178. v11. p8. Release date: December 18, 2019). The RNA sequencing read counts of sample were obtained from TCGA. All these samples were sequencing by Illumina Genome Analyzer IIX. According to the bioinformatics pipeline for mRNA analysis in TCGA (https://docs.gdc.cancer.gov/Data/Bioinformatics_Pipelines/Expression_mRNA_Pipeline/), quality assessment was performed with FASTQC (version 0.11.8), the alignment was performed using a two-pass method with STAR (version 2.4.2a). The reads were aligned to the GRCh38 reference genome and then were quantified with HTSeq (version 0.6.1). CPM normalization was performed to correct library size differences between samples. As an initial filter, we retained only genes with log_2_CPM >1 in more than half of the samples. To compare expression levels between samples, for these genes alone, we then re-normalised raw count data by TMM (implemented in edgeR version 3.32.1) and transformed these by voom in limma (version 3.46.0) (Robinson and Oshlack, 2010; McCarthy et al., 2012; Ritchie et al., 2015). After carrying out this normalization, mRNAs, microRNA and lncRNAs differentially expressed in tumor group and solid normal tissues were identified. Thresholds for differential expression were set each as the adjusted *p* value less than 0.01 and |log_2_fold change|>1.

### Construction of a lncRNA-miRNA-mRNA ceRNA Network

All of the differentially expressed genes were considered as candidates in the lncRNA-miRNA-mRNA ceRNA network construction, which was based on multiple factors such as binding affinity of miRNAs to their lncRNA sponges, RNA secondary structures and RNA-binding proteins, and the abundance and subcellular localization of ceRNA components (Qi et al., 2015). To construct the ceRNA network, the R package GDCRNATools (version 1.10.1) was used (Li et al., 2018). with five databases on miRNA-mRNA interactions including STarMir (version 2.2) (Kanoria et al., 2016), StarBase (version 2.0) (Li et al., 2014), miRcode (version 11) (Jeggari et al., 2012), spongeScan (version 1.0) (Furió-Tarí et al., 2016), and mirTarBase (version 7.0) (Chou et al., 2018) incorporated for the interactions analysis. The criteria for identifying competing lncRNA-mRNA interactions are: a) the strength of positive association between expression of lncRNA and its target mRNAs, b) the hypergeometric probability of shared miRNAs on the lncRNA-mRNA pair, c) the strength of regulation similarity of all shared miRNAs on the lncRNA-mRNA pair. Based on above criteria, *Pearson*’s correlation was used to measure the association between expression of lncRNA and mRNA. Hypergeometric distribution was measured by *Fisher*’s exact test. Regulation similarity was calculated based on the total number of the lncRNA-mRNA shared miRNAs, the *Pearson*’s correlation between the miRNA with lncRNA, as well as miRNA with mRNA (Li et al., 2018). After the construction, the ceRNA network was plotted using Cytoscape (version 3.7.0) software (Smoot et al., 2011).

### Survival Analysis

We investigated the prognostic values of the main differentially expressed lncRNAs in the ceRNA network for CRC patients. Kaplan-Meier curves for survival analysis were depicted to present the survival-related lncRNAs. A log-rank test was performed to compare the survival distributions of samples grouped by lncRNA differential expression levels of the patient cohort.

### Functional Annotation

To identify the biological function of the ceRNA network possibly contributing to tumor development, we performed a functional annotation analysis with multiple pathway databases, including Gene Ontology (GO) (Ashburner et al., 2000), Reactome (Haw et al., 2011), and Speed2 (Signalling Pathway Enrichment using Experimental Datasets 2) (Rydenfelt et al., 2020). A *p* value of less than 0.05 was considered to indicate statistically significant enrichment. The R package clusterProfiler V3.11 (Yu et al., 2012) was used for the GO and Reactome pathway enrichment analysis and visualization of significant modules. The R package SPEED2 was used for checking the upstream pathway activity from the genes in the ceRNA network, and a Bates test was used to calculate the test statistics for pathway enrichment.

### Optimization of the ceRNA Network

There were more than 100 genes in the ceRNA network (Figure 2), so for practical applicability, we confirmed the target structural accessibility and selected the critical lncRNAs associated with the survival of CRC patients as the hub genes. Only the mRNAs both correlated with the hub gene and in the original ceRNA network were used to reconstruct the optimized network. Target structural accessibility for miRNA target recognition was calculated and visualized using STarMirDB and Sfold (version 2.2) (Kanoria et al., 2016; Rennie et al., 2019). The optimized network was visualized using Cytoscape.

### Network Signature Analysis

Gene set variation analysis (GSVA) was used to determine the network expression level of each single sample, analogously to a competitive gene set test (Hänzelmann et al., 2013). By performing GSVA scoring, we were able to estimate the variation in the gene enrichments of the networks of the samples, and to do so independently for tumor and normal tissues. GSVA scores are designed to range from −1 to 1, with negative scores indicating relative decreases in network expression while positive scores indicated elevations. For practical applicability, only lncRNAs and mRNAs in networks were listed as the gene set signature for the GSVA assessment. The R package GSVA (v3.11) was used to calculate the GSVA score of networks over the samples in the CRC transcriptome dataset.

### Estimation of Immune Cell Infiltration

Immune cells infiltrated in the tumor microenvironment play crucial roles in tumor invasion and metastasis. To estimate immune cell infiltration in samples with different network expression levels, the web-based tool ImmuCellAI was applied to calculate the abundance of 24 immune cell subsets *via* their gene expression profiles (Miao et al., 2020). The immune cell subsets estimated in this study included 18 T cells subsets: CD4^+^T cells, CD8^+^T cells, naïve CD4^+^T cells, naïve CD8^+^T cells, natural regulatory T (nTreg) cells, induced regulatory T (iTreg) cells, gamma delta T (γδ T) cells, central memory T (Tcm) cells, effector memory T (Tem) cells, natural killer T (NKT) cells, T helper 1 (Th1) cells, T helper 2 (Th2) cells, and T helper 17 (Th17) cells, cytotoxic T (Tc) cells, exhausted T (Tex) cells, type 1 regulatory T (Tr1) cells, follicular T helper (Tfh) cells, mucosal-associated invariant T (MAIT) cells, and other six immune cell subests: B cells, natural killer (NK) cells, monocytes, macrophages, neutrophils and dendritic cells (DCs).

**FIGURE 1:**
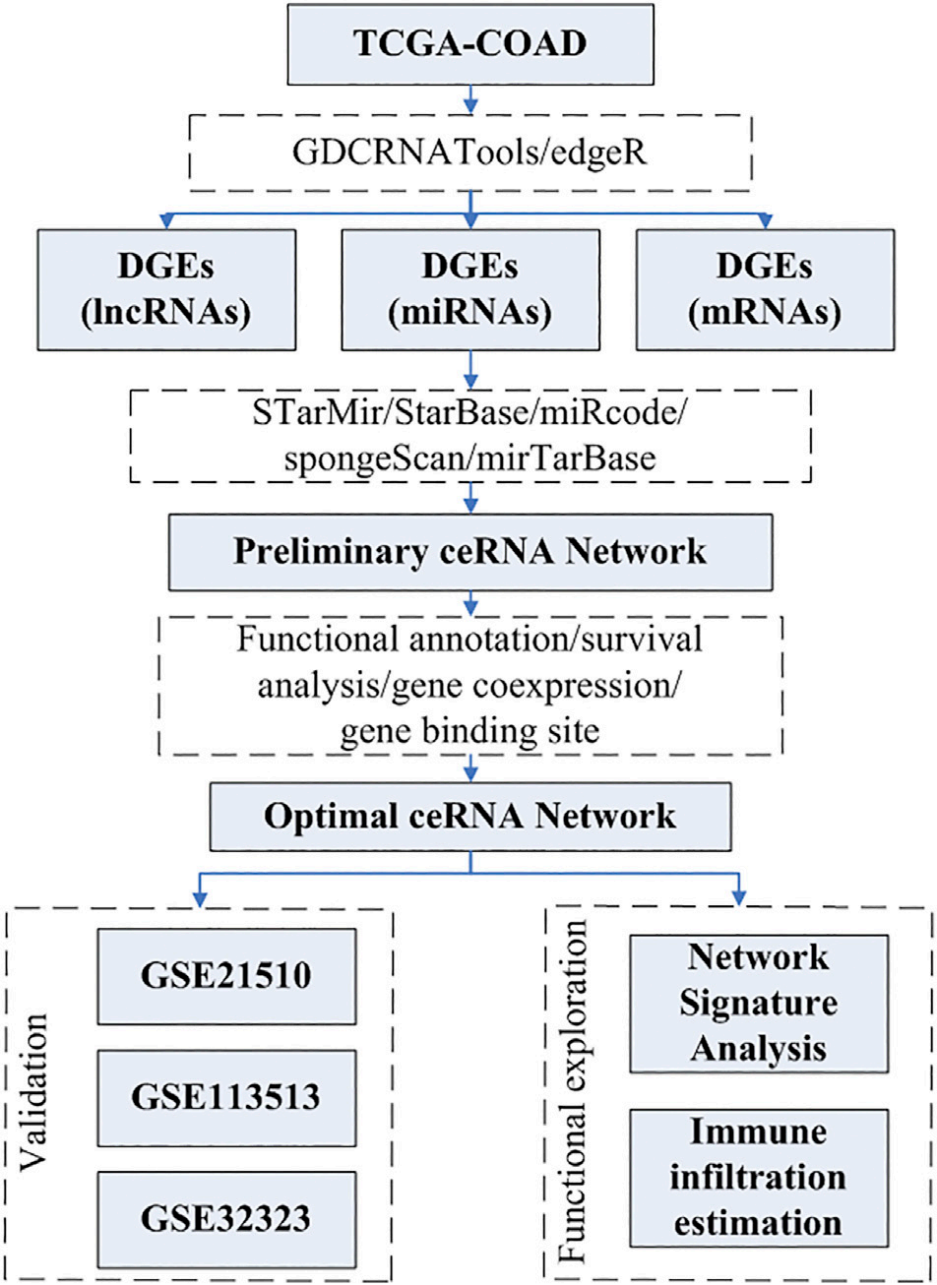
Flowchart of the research process.

The pipeline of this study is depicted in the flowchart shown in Figure 1.

## Results

### CRC Tumor Samples and Normal Samples Were Significantly Distinguished on the Basis of Differentially Expressed lncRNAs

We obtained the aligned read counts (GRCh38 (hg38) version) of tissue samples from 478 primary tumor, one metastatic tumor, one recurrent tumor and 41 normal solid tissue from TCGA. Samples from primary tumor, metastatic tumor and recurrent tumor were combined as tumor group. The age and sex of patients in normal group was matched to those in tumor group (Supplementary Table 1). There were total number of 60483 genes in this dataset. In prefiltering step, 14768 genes were kept for the downstream analyses. After TMM normalization and voom transformation, we explored the DGEs (differentially expressed genes) based on GLM (generalized linear model) likelihood ratio test, 2935 CRC tissue-specific mRNAs and 213 lncRNAs *via* the differential expression analysis of the TCGA-COAD dataset (Supplementary Figures S1A,B, Supplementary Table S2). The heatmaps of the differential expression of lncRNAs between CRC tissues and solid normal tissues are shown in Supplementary Figure S1C. The expression of 213 differentially expressed lncRNAs separated the tumor group and normal group clearly.

### Construction of the lncRNA-miRNA-mRNA ceRNA Network

**FIGURE 2:**
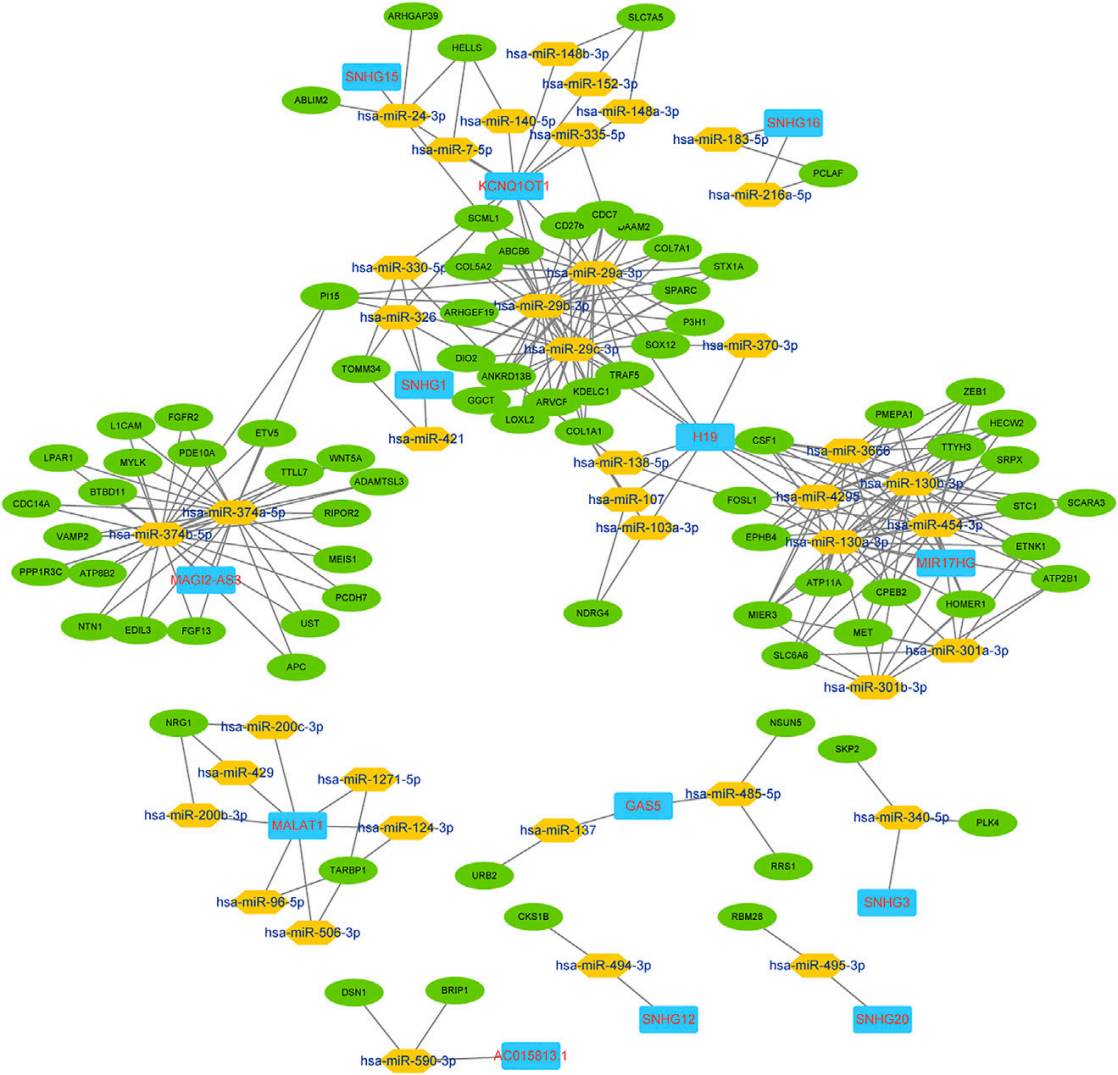
lncRNA-miRNA-mRNA ceRNA preliminary network.

In the current work, certain lncRNAs and mRNAs were shown to co-express in the ceRNA networks. We built the ceRNA network based on the co-expression patterns of the lncRNAs, miRNAs and mRNAs. In total, 133 nodes and 288 edges constituted the ceRNA network. The preliminary network is shown in Figure 2.

### Knowledge-Driven Pathway Analysis of ceRNA Network

**FIGURE 3:**
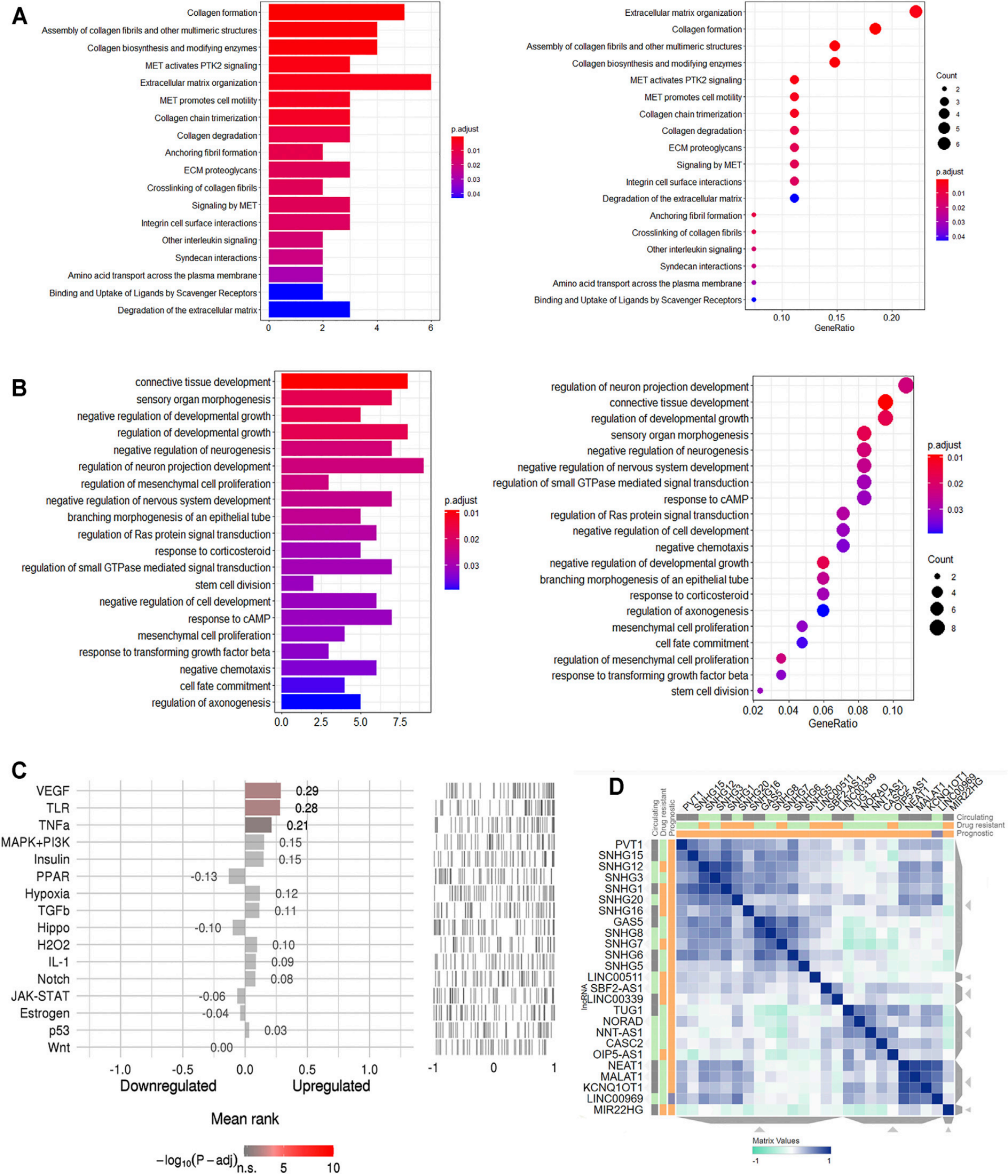
Functional annotation for the genes in ceRNA network of CRC. **(A)**. Reactome pathway enrichment of the genes in ceRNA network of CRC. **(B)**. The top 20 enriched molecular in GO terms. **(C)**. Speed2 pathway activity ranking. **(D)**. CRC related lncRNAs similarity.

According to the Reactome analysis, biological processes of the genes in the ceRNA network from tumor tissue were mainly related to MET pathways (Figure 3A). MET is a receptor tyrosine kinase (RTK), and like other related RTKs such as EGFR, MET can be activated by binding to its ligand, namely hepatocyte growth factor/scatter factor (HGF/SF), resulting in MET dimerization and *trans*-autophosphorylation.

A total of 106 GO terms were extracted using the GO analysis (Supplementary Table S3). Of the 106 GO terms, 97 were Biological Process terms, eight were Cellular Component terms and one was a Molecular Function term. The BP GO terms have all been confirmed to be related to the regulation of neurogenesis and mesenchymal cell proliferation (Figure 3B) with, for instance, GO:0050768 (negative regulation of neurogenesis), GO:0010975 (regulation of neuron projection development) and GO:0051961 (negative regulation of nervous system development) involving neurogenesis, and GO:0010464 (regulation of mesenchymal cell proliferation) and GO:0051591 (mesenchymal cell proliferation) involving proliferation of human mesenchymal cells.

SPEED2 analyses allow one to infer upstream pathways. In the current work, the VEGF pathway was indicated from this analysis to be the signaling pathway that likely caused the genes in the ceRNA network to be deregulated (Figure 3C, Supplementary Table S4). We used another web-based tool LnCeVar-Cluster which provides cluster profiles between differential expressed lncRNAs and ceRNAs, especially the similarity profiles between differential expressed lncRNAs (Wang et al., 2020). The differential expressed lncRNAs clustering profile of TACG-COAD indicated that NEAT1, MALAT1, KCNQ1OT1 and LINC00969 had the similar expression pattern in CRC (Figure 3D). Functional annotations showed the ceRNA network genes to be closely related to the tumor pathogenesis in general.

### KCNQ1OT1 Determined to Be the Critical Hub Gene for the Network Optimization

**FIGURE 4:**
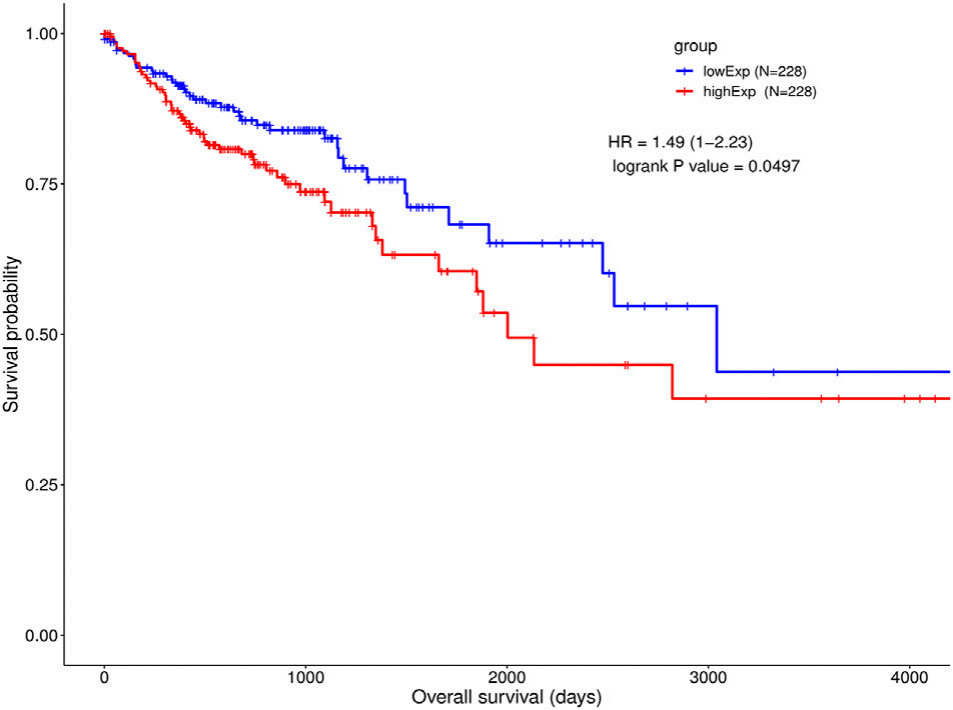
Survival analysis of KCNQ1OT1 ceRNA network and gene co-expressions. Kaplan-Meier and ROC curves of CRC cohort in high and low KCNQ1OT1 expression level.

We chose the hub lncRNAs (degree >5) and their related miRNAs and mRNAs in the ceRNA network. We identified 22 lncRNAs involved in preliminary ceRNA networks (MIER3, MET, ANKRD13B, ATP11A, TTYH3, EPHB4, FOSL1, MIR17HG, TARBP1, COL1A1, SCML1, ATP2B1, H19, CPEB2, PMEPA1, KCNQ1OT1, SOX12, CDC7, HECW2, MALAT1, CSF1, PI15) (Figure 2). Then we analyzed the association of the hub lncRNAs with the clinically obtained survival data to identify the lncRNAs crucial to CRC prognosis. Only the lncRNA KCNQ1OT1 was significantly differentially expressed according to the log-rank test of survival analyses (Table 1). A Kaplan-Meier estimate showed poorer prognoses for patients with higher levels of KCNQ1OT1 (Figure 4).

**TABLE 1:** Survival analysis with the critical hub genes.

lncRNAs	HR	Lower 95	Upper 95	*p* value
MIER3	0.93122	0.62377	1.39021	0.726
MET	0.820336	0.549485	1.224696	0.331
ANKRD13B	1.255134	0.841125	1.872922	0.265
ATP11A	1.148337	0.769663	1.713318	0.498
TTYH3	1.224092	0.820421	1.826383	0.322
EPHB4	1.009199	0.676432	1.505668	0.964
FOSL1	1.160118	0.77757	1.730871	0.467
MIR17HG	1.021937	0.684606	1.525483	0.915
TARBP1	1.066937	0.714761	1.592637	0.751
COL1A1	1.112393	0.74518	1.660564	0.601
SCML1	0.95713	0.641016	1.429135	0.829
ATP2B1	0.920591	0.616832	1.373935	0.683
H19	1.108367	0.742886	1.653656	0.614
CPEB2	0.77813	0.520825	1.162552	0.217
PMEPA1	0.999453	0.669902	1.491122	0.998
KCNQ1OT1	1.493465	1.000447	2.229441	0.0497*
SOX12	1.331673	0.892524	1.986897	0.161
CDC7	1.131191	0.757923	1.688289	0.547
HECW2	1.059556	0.710069	1.581057	0.776
MALAT1	1.416188	0.948783	2.113852	0.0881
CSF1	1.324722	0.886839	1.978812	0.166
PI15	1.039925	0.696976	1.551622	0.847

**FIGURE 5:**
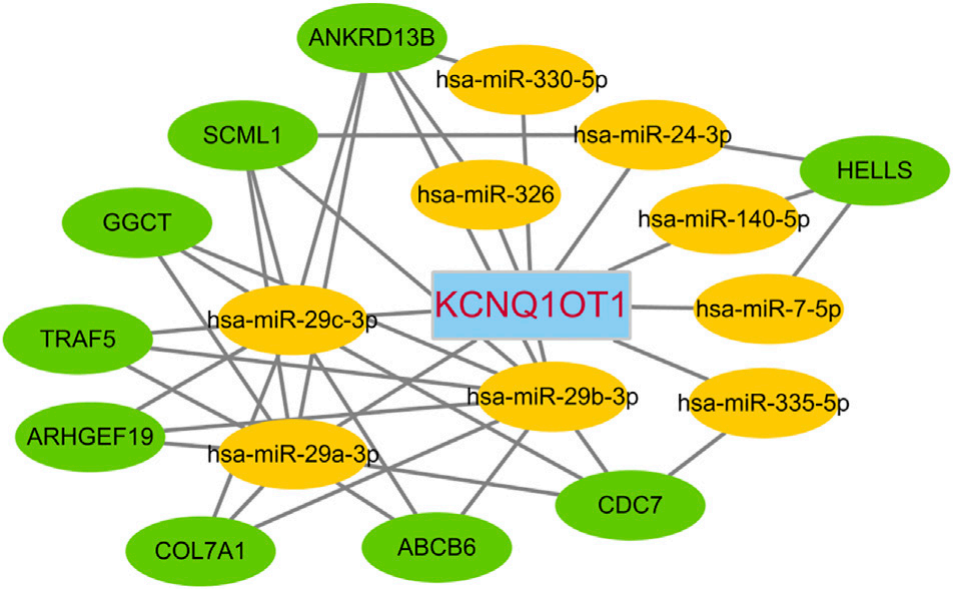
KCNQ1OT1 ceRNA network.

**FIGURE 6:**
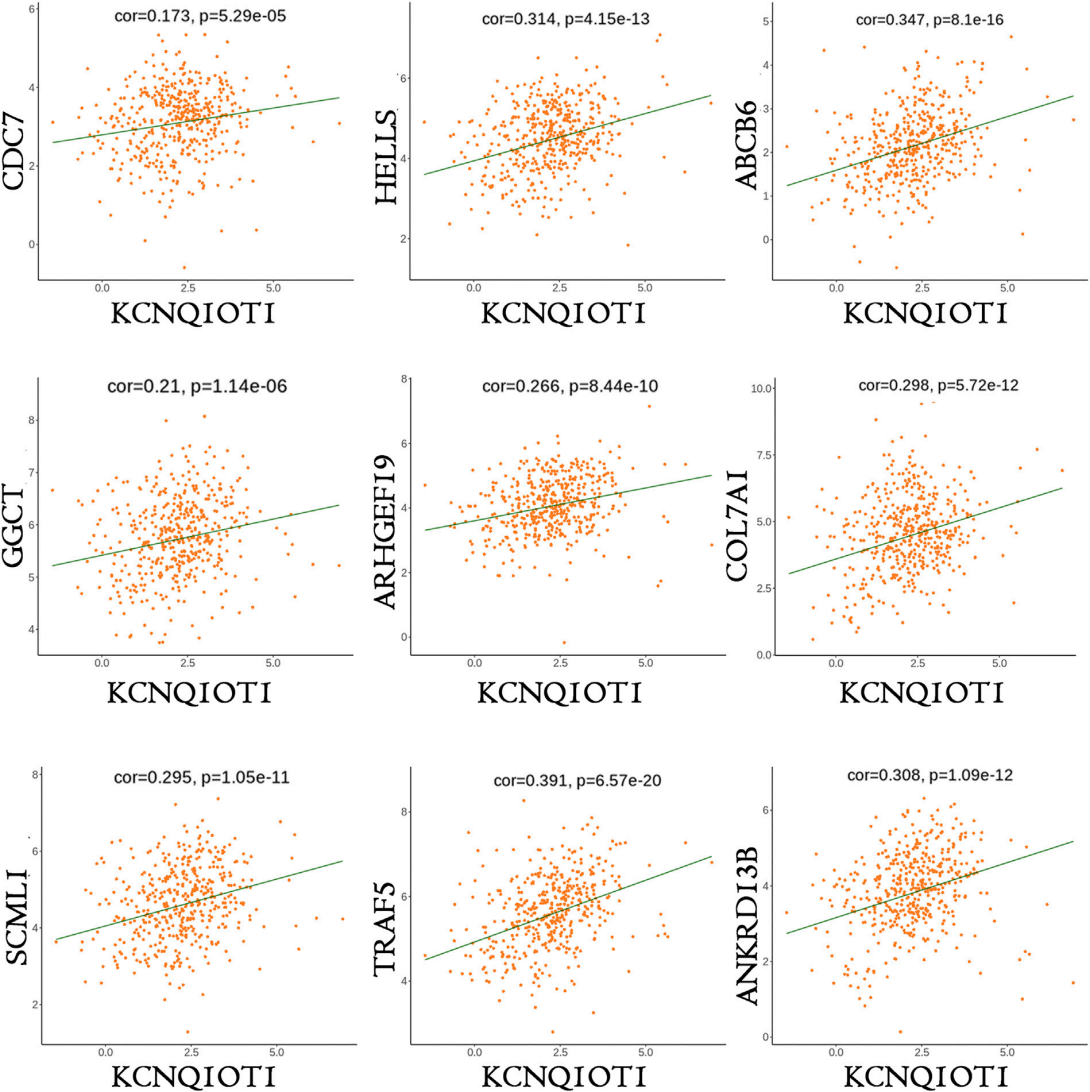
Co-expressions of mRNAs and KCNQ1OT1 in KCNQ1OT1 ceRNA network.

Of the lncRNAs present in the preliminary ceRNA network, only one gene, namely KCNQ1OT1, was indicated to be associated with survival in the CRC cohort. Thus, a correlation analysis was performed based on this critical KCNQ1OT1 hub gene. Based on a hypergeometric test and correlation analysis (Table 2), the mRNAs and lncRNAs not significantly correlated with KCNQ1OT1 or without a direct link with KCNQ1OT1 were removed from the network. The KCNQ1OT1-miRNA-mRNA subnetwork consisted of one centroid lncRNA node, nine miRNA nodes and nine mRNA nodes. This KCNQ1OT1 ceRNA network was reconstructed and visualized using Cytoscape (Figure 5). The expression level of the lncRNA KCNQ1OT1 was positively correlated with the expression levels of the nine mRNAs (Figure 6). This result was consistent with the ceRNA theory that the lncRNA regulated other RNA transcripts by competing for shared microRNAs. We checked the expression profile of KCNQ1OT1 and other hub network genes in tumor group and normal group in TCGA data set (Supplementary Table S2, Supplementary Figure S2). An additional searching in The Genotype-Tissue Expression (GTEx) portal (dbGaP accession number phs000424. vN.pN) which is a comprehensive public dataset for tissue-specific gene expression and regulation in non-diseased cohort for verification of the expression of KCNQ1OT1 in colon was conducted (Supplementary Figure S3). The results showed that the gene expression of KCNQ1OT1 in both transverse colon and sigmoid colon was higher than in blood. And the gene expression of KCNQ1OT1 across all tissues showed that, although KCNQ1OT1 did not represent the highest expression in colon, its expression level was not low either, consistent with what we found in TCGA-COAD: the KCNQ1OT1 expression in normal tissue was low (CPM: median: 2.41, mean: 3.46). But its expression level in tumor tissue in TCGA-COAD was higher (CPM: median: 4.73, mean: 10.67). All this data showed that KCNQ1OT1 had enough expression to physiologically function as a miRNA sponge.

**TABLE 2:** Hypergeometric test and Correlation analysis for mRNAs selection.

Genes	Fold enrichment	Hyper *P*	Shared miRNAs	Cor R	Cor *P*
ANKRD13B	3.763586957	0.00598274	hsa-miR-29c-3p,hsa-miR-29b-3p,hsa-miR-326,hsa-miR-330–5p,hsa-miR-29a-3p	0.192288435	2.05E-05
COL7A1	6.02173913	0.008538913	hsa-miR-29c-3p,hsa-miR-29b-3p,hsa-miR-29a-3p	0.204474578	6.30E-06
HELLS	9.032608696	0.001910986	hsa-miR-7-5p,hsa-miR-140–5p,hsa-miR-24–3p	0.231966962	3.36E-07
TRAF5	12.04347826	0.000505415	hsa-miR-29c-3p,hsa-miR-29b-3p,hsa-miR-29a-3p	0.331376549	2.86E-13
SCML1	4.817391304	0.005491359	hsa-miR-29c-3p,hsa-miR-29b-3p,hsa-miR-24–3p,hsa-miR-29a-3p	0.234030918	2.65E-07
ARHGEF19	12.04347826	0.000505415	hsa-miR-29c-3p,hsa-miR-29b-3p,hsa-miR-29a-3p	0.197882747	1.20E-05
GGCT	12.04347826	0.000505415	hsa-miR-29c-3p,hsa-miR-29b-3p,hsa-miR-29a-3p	0.14387579	0.001121
ABCB6	12.04347826	0.000505415	hsa-miR-29c-3p,hsa-miR-29b-3p,hsa-miR-29a-3p	0.294248362	1.02E-10
CDC7	4.37944664	0.008147588	hsa-miR-29c-3p,hsa-miR-29b-3p,hsa-miR-335–5p,hsa-miR-29a-3p	0.113130038	0.008238

Target structural accessibility for miRNA target recognition to lncRNA KCNQ1OT1 or mRNAs was calculated and visualized using Sfold and STarMirDB (Supplementary Figures S4, S5).

### The KCNQ1OT1 ceRNA Network Signature Was Highly Expressed in CRC Transcriptomic Profiles

**FIGURE 7:**
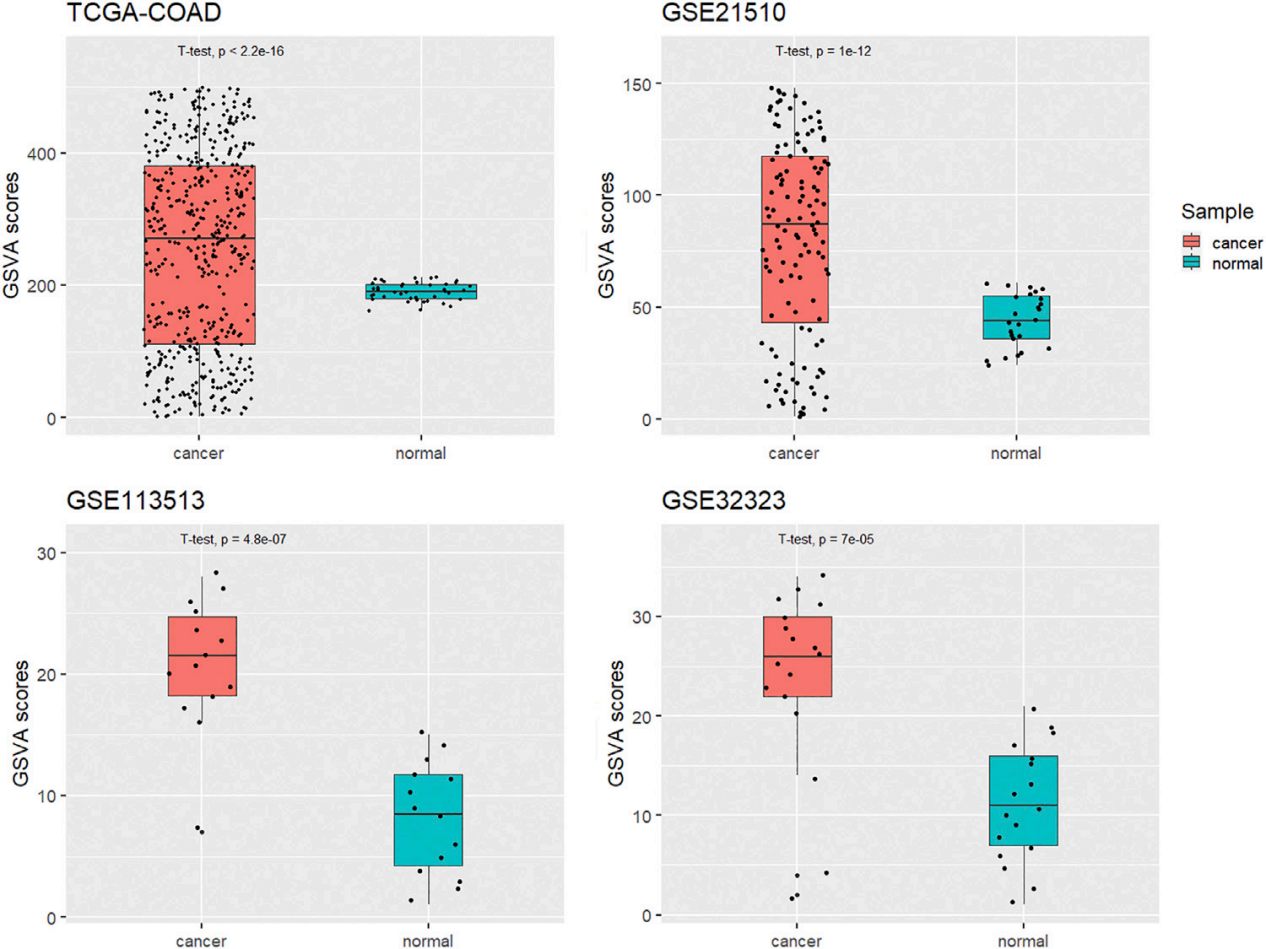
GSVA scores of KCNQ1OT1 ceRNA network for cancer and normal samples in four different CRC datasets.

A GSVA assessment of network signatures in tumor and normal samples and using the TCGA-COAD dataset showed higher network signature GSVA scores for the tumor tissues than for the normal samples. This result was expected because we constructed this network from DEGs in the TCGA-COAD dataset. For validation, we applied the GSVA assessment of network signatures in three GEO CRC transcriptome datasets (GSE21510, GSE113513 and GSE32323). In these GEO datasets, KCNQ1OT1 ceRNA network signature GSVA scores were significantly higher for tumor samples than for normal samples, which indicated the disease specificity of this KCNQ1OT1 ceRNA network (Figure 7).

### Different Immune Cell Infiltration Levels for High- and Low-GSVA-Score Tumor Samples

**FIGURE 8:**
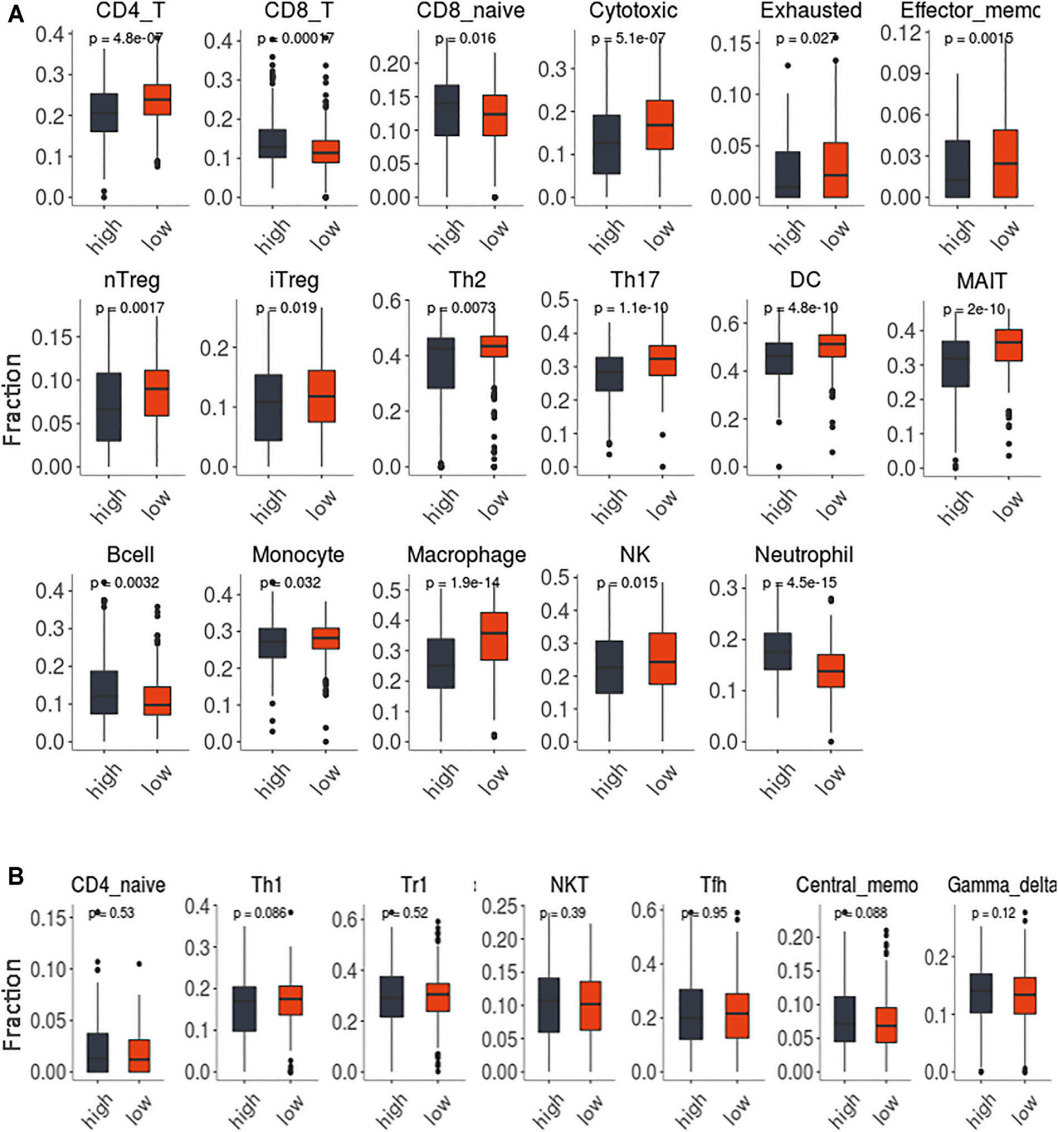
The immune infiltration of 24 immune cells subsets in the tumor samples with high GSVA score and low scores. **(A)**. The comparisons with statistically significant difference. **(B)**. The comparisons without statistically significant difference.

The immune cell infiltration levels for 24 immune cell subsets in tumor samples from the TCGA-COAD dataset are shown in Figure 8. Overall, the profiles of immune infiltration varied significantly between the tumor samples with high GSVA scores and those with low scores (Figure 8A). Also, lower CD4^+^/CD8^+^ T-cell ratios were found in the tumor samples with high network GSVA scores than in the tumor samples with low scores. In the high-score group, along with the increase in the levels of CD8^+^ T cells in general was observed an increase in those of naïve CD8^+^ T cells. Notably, in contrast, lower levels of cytotoxic T cells were found for the high-score group than for the low-score group. Also, along with the relatively low levels of CD4^+^ T cells in the tumor samples with high scores, were relatively low levels of the T helper subsets Th2 and Th17. Other T cell subsets, namely Treg (nTreg and iTreg), Tex, Tem and MAIT cells were also downregulated. However, the B cells were upregulated. Regarding another lymphoid cell line, natural killer cell levels showed relatively low levels for the high-score group. Further, regarding the myeloid cell line, lower levels of monocytes, macrophages and neutrophils were also found for the high-score group.

Patient sex, tumor stage and age were also investigated. However, the network GSVA score was neither associated with sex (*p* = 0.8) nor with age (*p* = 0.77) nor with tumor stage (*p* = 0.78), as shown in Supplementary Figures S6A–C, respectively.

## Discussion

There has been increasing evidence for a link between dysregulation of lncRNAs and cancers (Gutschner and Diederichs, 2012). For instance, studies have shown the lncRNA MALAT1 to be associated with the development and metastasis of cancer cells (Gutschner et al., 2013; Tripathi et al., 2013). HOTAIR to be implicated in cancer metastasis regulation by targeting the chromatin repressor polycomb protein (Gupta et al., 2010), and linc00673 to activate WNT/β-catenin signaling and aggravate lung adenocarcinoma by binding between casein kinase 1ε (CK1ε) and DEAD box RNA helicase DDX3 (Guan et al., 2019).

Recent studies have also demonstrated important roles played by lncRNAs in the tumorigenesis of CRC. Colorectal cancer associated transcript 1 (CCAT1) was reported to be a specific biomarker for CRC (Xiang et al., 2014), and to be expressed at high levels not only in pre-malignant conditions but also throughout the various disease stages of CRC (Ozawa et al., 2017). Further, CCAT2, a lncRNA derived from the human chromosome MYC-335 region, has been shown to enhance metastasis and invasion through the MYC pathway-regulating miRNAs miR-20a29 and miR-17–5p. These two CRC-specific lncRNAs were shown to be transcribed from the 8q24 region where previous studies have found common genetic variants related to the risk of CRC (Yang et al., 2019).

In the current work, we showed that the lncRNA KCNQ1OT1, an antisense lncRNA transcribed from the human chromosome 11p15.5 KCNQ1 locus, is related to the pathogenesis and progression of CRC. It has been shown in previous work to function as a *cis*-silencer of the imprinted KCNQ1 cluster and to be involved in the metastasis and proliferation of various tumors, such as hepatocellular carcinoma, cholangiocarcinoma, ovarian and breast cancer tumors (Feng et al., 2018; Luo and Jin, 2019).

In our research, there were 213 lncRNAs differentially expressed between tumor and normal tissue. Among these 213 lncRNAs, we identified 22 lncRNAs involved in ceRNA networks. We analyzed the survival association with these 22 lncRNAs. Only KCNQ1OT1 significantly associated with the clinically obtained survival data. We compared 228 patients expressing KCNQ1OT1 at high levels with 228 patients expressing it at low levels. According to our Kaplan-Meier survival analysis, the patients with overexpressed KCNQ1OT1 did not survive on average for as long as did those displaying low KCNQ1OT1 expression. This result was consistent with the latest research (Lin et al., 2021). KCNQ1OT1 was reported to be upregulated in CRC tissue according to a study by Li and others (Li F. et al., 2019). KCNQ1OT1 knockdown in HCT116 and SW480 CRC cells downregulated the expression of Atg4B, which has been shown to cleave LC3 and promote the formation of autophagosomes (Hemelaar et al., 2003). The viability of these cells decreased after being treated with oxaliplatin, which implicated KCNQ1OT1 in inducing autophagy protection and chemo-resistance. Moreover, the relationship between the upregulation of KCNQ1OT1 and poor prognosis of CRC patients also suggested that higher KCNQ1OT1 levels in patients make them resistant to chemotherapy or other anti-cancer treatments (Li Y. et al., 2019). These results suggested that KCNQ1OT1 might become a promising therapeutic target for use in CRC patients.

The development of an understanding of RNA-RNA interactions is expected to provide deep insights into gene regulatory networks potentially implicated in various cancer diseases. Our optimized ceRNA network showed that the critical hub gene KCNQ1OT1 indirectly regulated 9 other mRNAs, namely those encoding ATP-binding cassette subfamily B member 6 (ABCB6), Rho guanine nucleotide exchange factor 19 (ARHGEF19), helicase lymphoid-specific (HELLS), gamma-glutamylcyclotransferase (GGCT), cell division cycle 7 (CDC7), sex comb on midleg-like 1 (SCML1), collagen type VII alpha 1 chain (COL7A1), TNF-receptor-associated factor 5 (TRAF5) and ankyrin repeat domain 13B (ANKRD13B) (Figure 5). According to our correlation analysis, these 9 mRNAs showed a co-expression relationship with KCNQ1OT1. A GeneCards (www.genecards.org) (Stelzer et al., 2016) analysis showed these gene products to be involved in some cancer-related pathways, for instance TRAF5 in the RANKL/RANK (receptor activator of NFKB (ligand)) signaling pathway, COL7A1 in ERK signaling, HELLS in the AMPK enzyme complex pathway and BRCA1 pathway, ARHGEF19 in RET signaling, and GGCT in a cancer metabolism pathway. These mRNAs and KCNQ1OT1 influence each other’s level by competing for the same pool of microRNAs: miR-29c-3p, miR-29b-3p, miR-326, miR-330-5p, miR-29a-3p, miR-7-5p, miR-140-5p, miR-24-3p and miR-335-5p. Among these microRNAs, the binding sited of miR-7-5p, miR-29a-3p, miR-29c-3p, miR-140-5p, miR-326 and hsa-miR-335-5p with KCNQ1OT1 have been confirm by experimental studies (Hu et al., 2018; Sun et al., 2018; Cheng et al., 2020; Mu et al., 2020; Yao et al., 2020; Zhou et al., 2021).

As the GSVA method can be used to score a gene set signature and depends neither on the composition nor size of a dataset, we applied this method to measure the optimal network signature across different datasets. Samples with a high network score across the independent datasets were found, based on the results, to be particularly enriched in the CRC tumor group.

The identified subnetwork was reported previously to be significantly more reproducible between different disease cohorts then were individual marker genes (Chuang et al., 2007), and in our study, the results of all these datasets indicated dramatically higher network scores for the tumor samples than for the normal samples (Figure 7). These results suggested that the KCNQ1OT1 ceRNA network might play a role in the mechanism of CRC pathogenesis. This network signature could be a potentially way to distinguish CRC tissue form normal tissue.

To gain more knowledge about the function of the KCNQ1OT1 ceRNA network related to immune infiltration, we used ImmuCellAI to estimate the abundance of immune cells from individual samples. The estimation implied that the overall profiles of immune infiltration differed significantly between the tumor samples with high GSVA scores and those with low scores (Figure 8A, Supplementary Table S7, S8). Lower CD4^+^/CD8^+^ T-cell ratio s were indicated for the higher-score groups. Both the reduction of the CD4^+^ T cell population and increase of the CD8^+^ T cell population contributed to the lower ratio of CD4^+^/CD8^+^ T-cell. The CD4^+^/CD8^+^ T-cell ratio was considered as an immunostimulatory marker in the general population (Gojak et al., 2019). A low CD4^+^/CD8^+^ T-cell ratio has been shown to be an immune risk phenotype related to chronic inflammation, persistent immune dysfunction and immune senescence (Wikby et al., 2005). In some investigations, the CD4^+^/CD8^+^ T-cell ratio was described as a significant marker for prognostic prediction in HIV/AIDS patients (Castilho et al., 2019; Gojak et al., 2019; F; Li F. et al., 2019). Notably, in our study, the trend found for CD8^+^ T cells was opposite that found for cytotoxic T cells (cytokine-produced CD8^+^ T cells) in the network score comparisons. The generation of a functional cytotoxic T-cell response in general depends on the activation of Th1 cells. However, in the current work, the proportions of Th1 cells in the high- and low-GSVA-score groups showed no significant difference. Another possibility was that a recruitment of CD8^+^ T cells in the tumor tissue accompanied the reduction of cytotoxic T cells, and that this recruitment reflected a suppression of the cytotoxic machinery of the infiltrates, suggesting that the dysfunctional status of the effector cells was due to the microenvironment in the samples with high network scores.

Beside the immune infiltration, the network score differences in sex, tumor stage and age were also investigated. Neither any sex bias nor age association with the network scoring was found according to the statistical analysis (Supplementary Figure S6).

Our findings investigated a network signature for the lncRNA KCNQ1OT1, which was shown in the current work to be a representative of the ceRNA network transcribed in CRC tissues. This ceRNA network could be a potential regulator in colorectal cancer immunity. These findings indicated an oncogenic role for KCNQ1OT1 and its downstream target mRNAs in the pathogenesis and progression of CRC.

## Conclusion

The results of our study indicated that the KCNQ1OT1 ceRNA network could be involved in the regulation of the CRC tumor microenvironment, providing a rationale for the further exploitation of KCNQ1OT1 as a possible functional contributor to and therapeutic target for CRC.

## Data Availability

The original contributions presented in the study are included in the article/Supplementary Material, further inquiries can be directed to the corresponding author.

## Glossary

ABCB6: ATP binding cassette subfamily B member 6
ANKRD13B: ankyrin repeat domain 13B
ARHGEF19: rho guanine nucleotide exchange factor 19
BP: biological process
CC: cellular component
CCAT1: colorectal cancer associated transcript 1
CDC7: cell division cycle 7
COL7A1: Collagen Type VII Alpha 1 Chain
ceRNA: competitive endogenous RNA
CK1ε: casein kinase 1ε
CRC: colorectal cancer
DC: dendritic cell
DEGs: differentially expressed genes
γδ T: gamma delta T cell
GGCT: gamma-glutamylcyclotransferase
GO: gene ontology
GSVA: gene set variation analysis
GTEx: The Genotype-Tissue Expression
HELLS: helicase, lymphoid-specific
HGF: hepatocyte growth factor
iTreg: induced regulatory T cell
KM: Kaplan-Meier
lncRNAs: long non-coding RNAs
MAIT: mucosal-associated invariant T cell
MF: molecular function; miRNA, microRNA
ncRNAs: non-coding RNAs
NK: natural killer cell
NKT: natural killer T cell
nTreg: natural regulatory T cell
RANKL/RANK: receptor activator of NFKB (ligand)
RTK: receptor tyrosine kinase
SCML1: sex comb on midleg-like 1 (Drosophila)
SF: scatter factor
siRNAs: small interfering RNA
Speed2: Signalling Pathway Enrichment using Experimental Datasets 2
Tc: cytotoxic T cell
TCGA: The Cancer Genome Atlas
Tcm: central memory T cell
Tex: exhausted T cell
Tem: effector memory T cell
Tfh: follicular T helper cell
Th1: T helper 1 cell
Th2: T helper 2 cell
Th17: T helper 17 cell
TMM: trimmed mean of M values
TRAF5: TNF receptor associated factor 5
Tr1: type 1 regulatory T cell.
