# Supplementary figures and images for: Regulation of Long Non-coding RNA KCNQ1OT1 Network in Colorectal Cancer Immunity
Source: Front Genet. 2021 Sep 22;12:684002. doi: 10.3389/fgene.2021.684002 (PMC8493092; doi:10.3389/fgene.2021.684002)

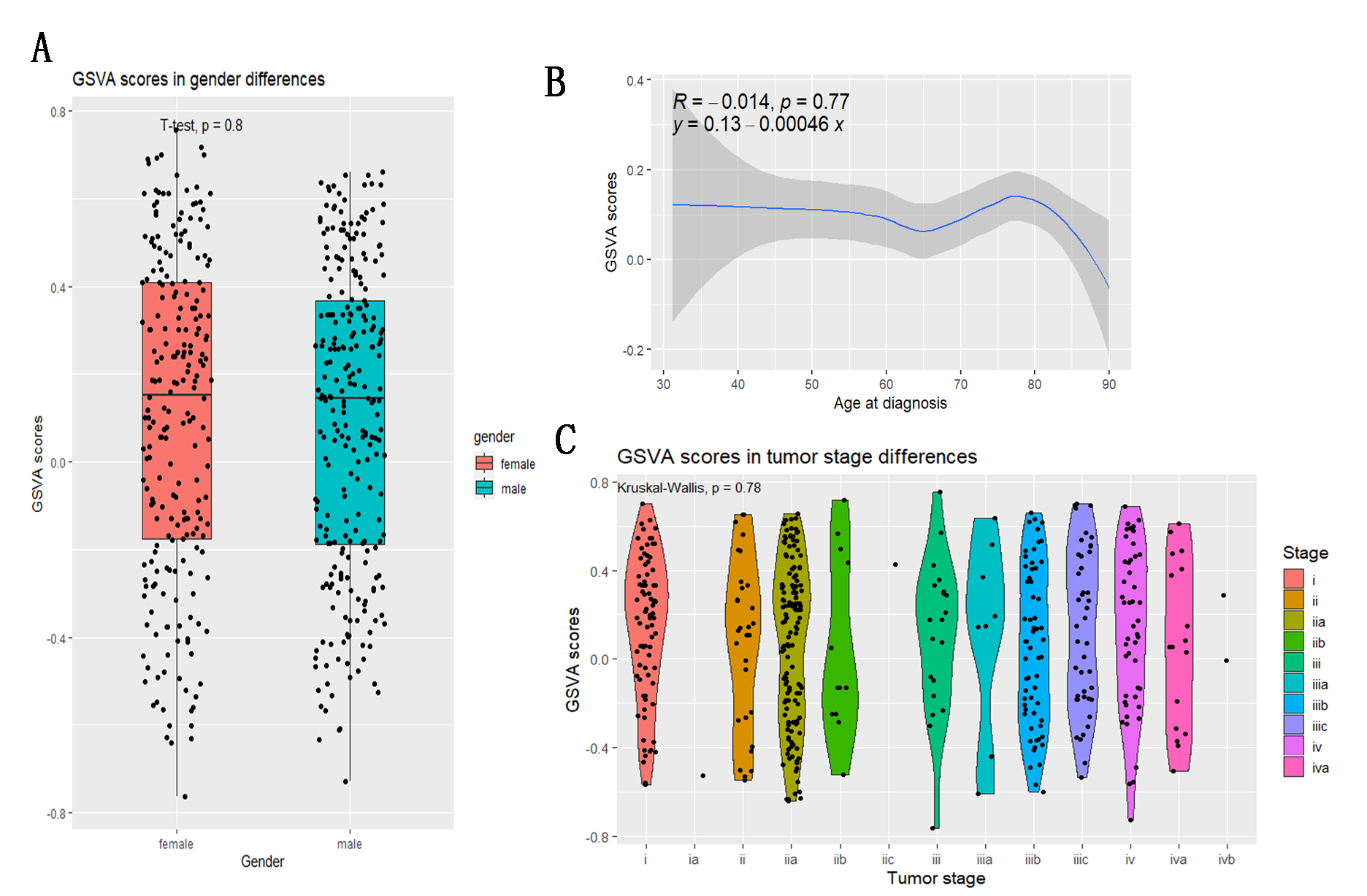

Supplement: Supplementary file 1 [file Image6.TIF]

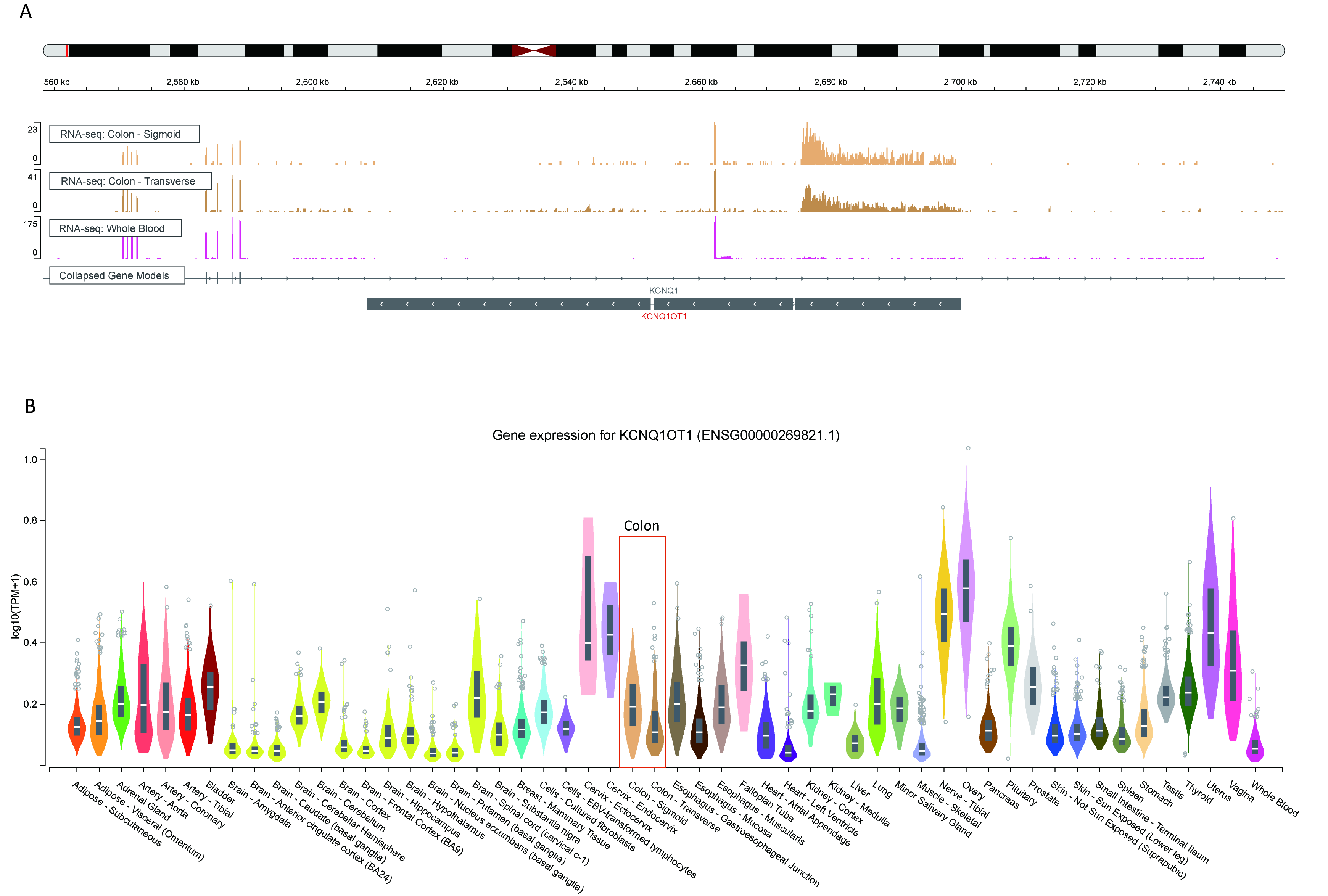

Supplement: Supplementary file 2 [file Image3.TIF]

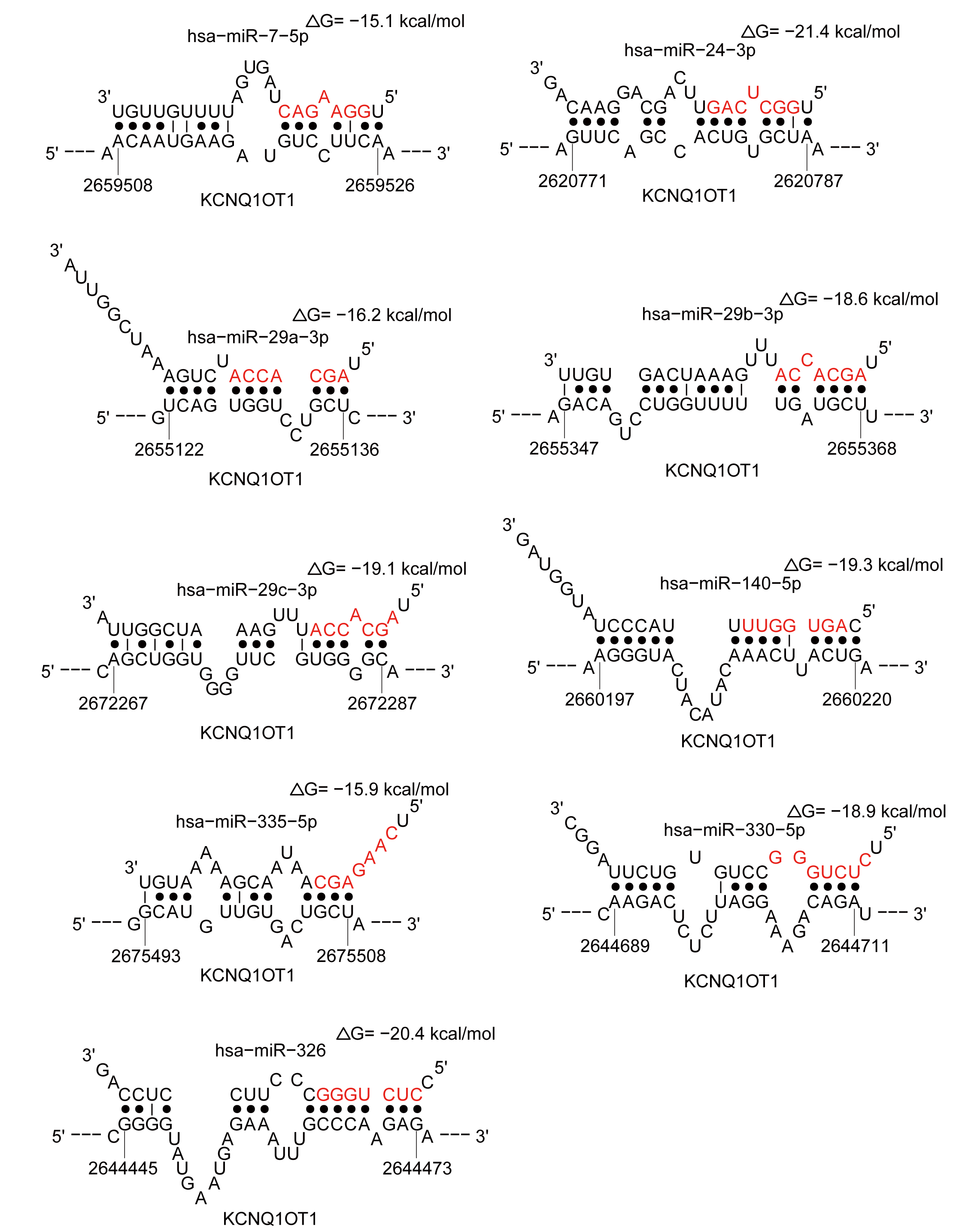

Supplement: Supplementary file 3 [file Image4.TIF]

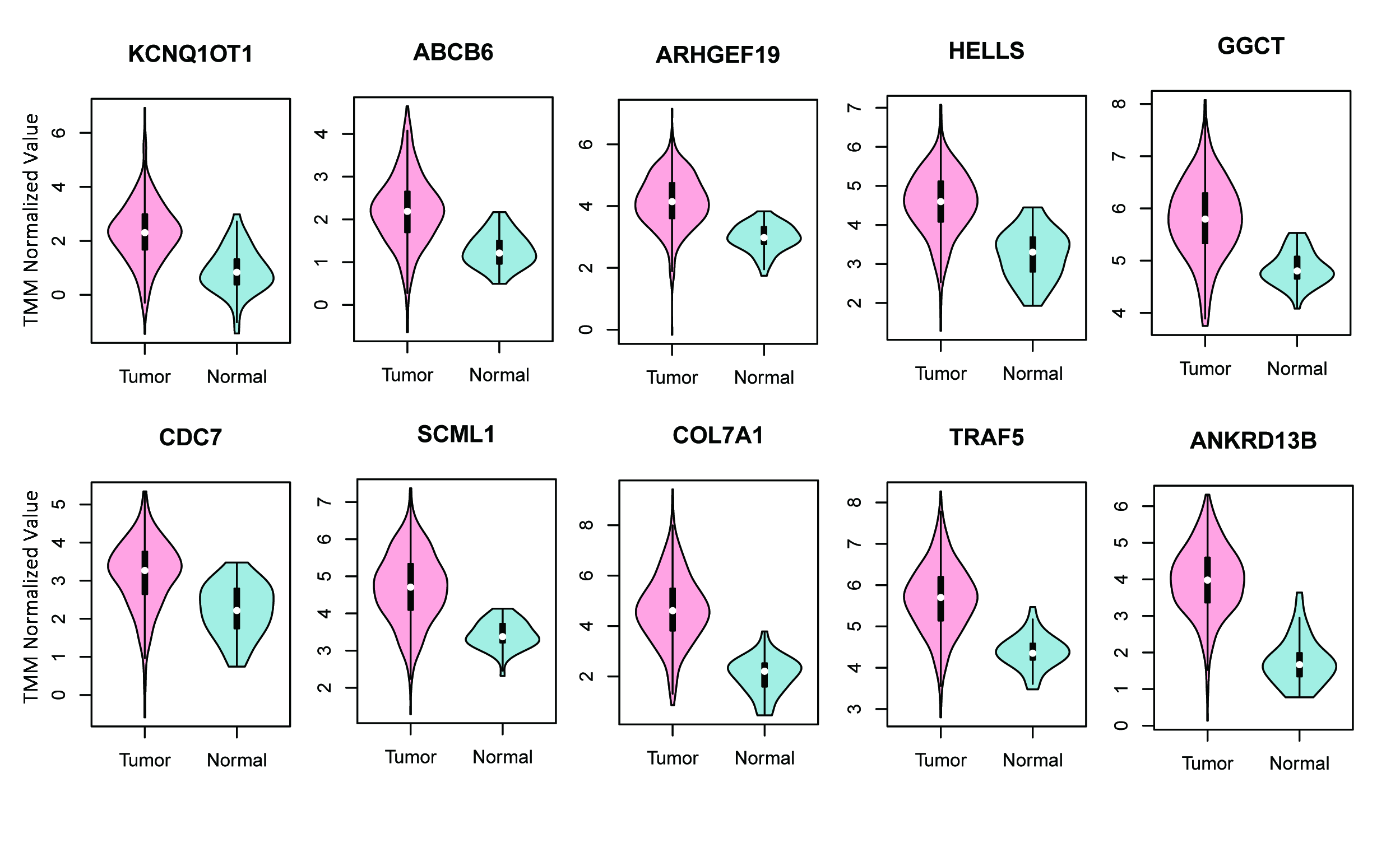

Supplement: Supplementary file 4 [file Image2.TIF]

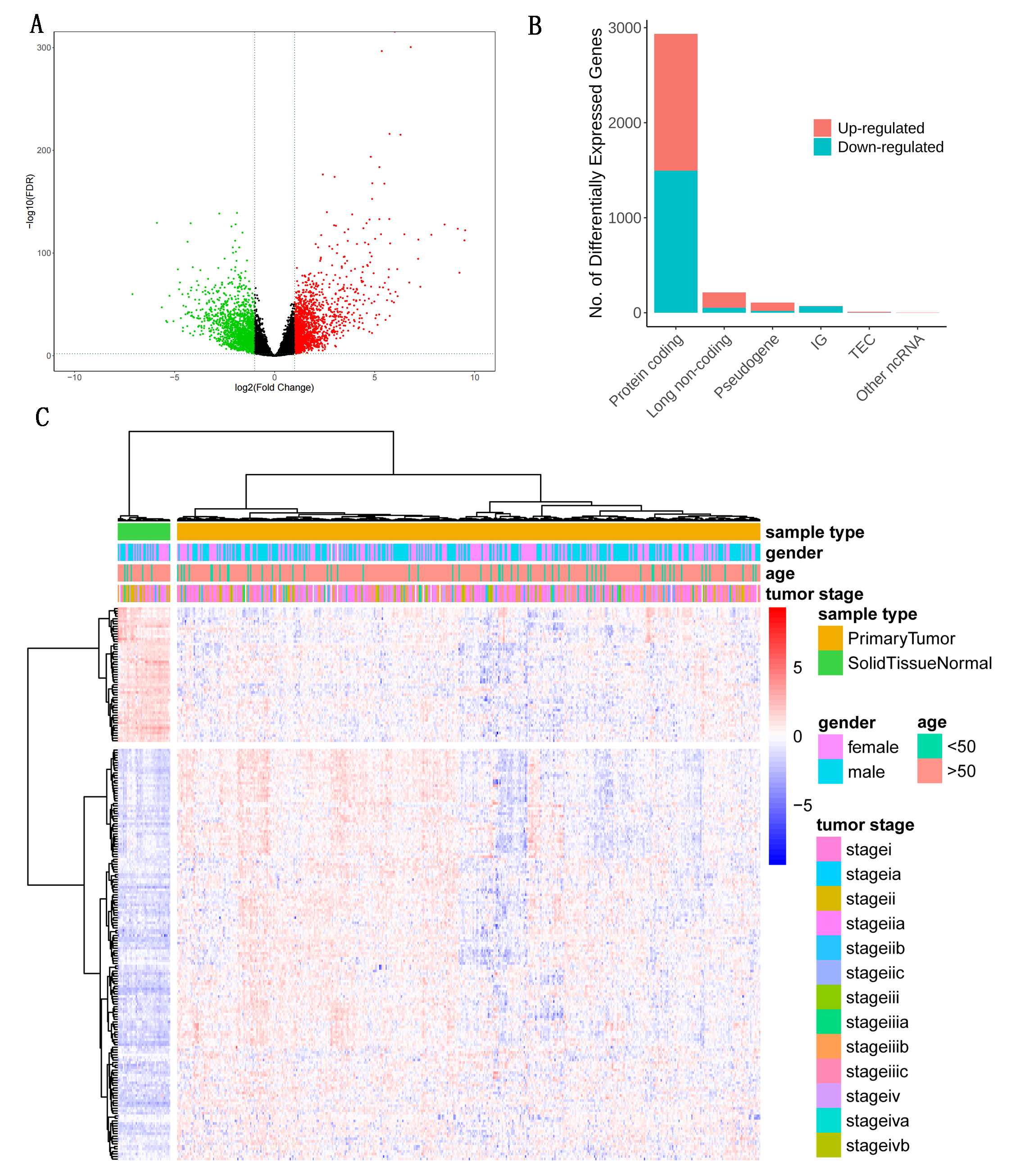

Supplement: Supplementary file 5 [file Image1.TIF]

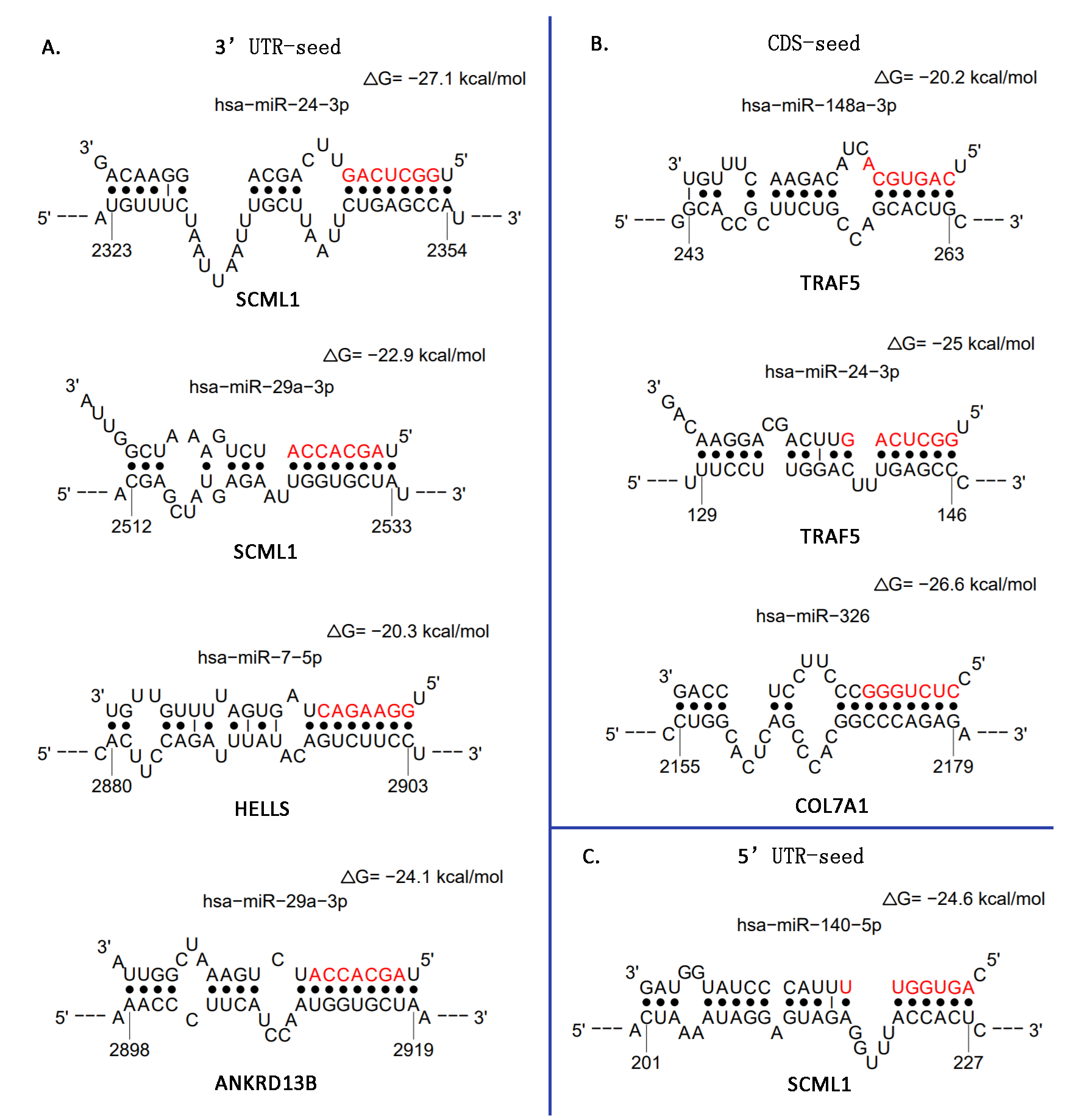

Supplement: Supplementary file 6 [file Image5.TIF]
